# Identification and expression analysis of the *WRKY* gene family during different developmental stages in *Lycium ruthenicum* Murr. fruit

**DOI:** 10.7717/peerj.10207

**Published:** 2020-10-28

**Authors:** Richard John Tiika, Jia Wei, Rui Ma, Hongshan Yang, Guangxin Cui, Huirong Duan, Yanjun Ma

**Affiliations:** 1 College of Forestry, Gansu Agricultural University, Lanzhou, Gansu Province, China; 2 Lanzhou Institute of Husbandry and Pharmaceutical Science, Chinese Academy of Agricultural Sciences, Lanzhou, Gansu Province, China

**Keywords:** *Lycium ruthenicum*, WRKY gene, Fruit developmental stages, Gene expression, Conserved motif, Transcriptome data

## Abstract

**Background:**

The* WRKY* gene family, one of the major transcription factor families in plants, plays crucial regulatory roles in physiological and biological developmental processes, and the adaptation of plants to the environment. However, the systematic study of WRKY structure, expression profiling, and regulatory functions has not been extensively reported in *Lycium ruthenicum*, although these aspects have been comprehensively studied in most plant species.

**Methods:**

In this study, the *WRKY* genes were identified from a *L. ruthenicum* transcriptome database by using bioinformatics. The identification, phylogenetic analysis, zinc-finger structures, and conserved motif prediction were extensively explored. Moreover, the expression levels of 23 selected genes with fragments per kilobase of exons per million mapped reads (FPKM) >5 were assayed during different fruit developmental stages with real-time quantitative polymerase chain reaction (RT-qPCR).

**Results:**

A total of 73 putative WRKY proteins in the *L. ruthenicum* transcriptome database were identified and examined. Forty-four proteins with the WRKY domain were identified and divided into three major groups with several subgroups, in accordance with those in other plant species. All 44 LrWRKY proteins contained one or two conserved WRKY domains and a zinc-finger structure. Conserved motif prediction revealed conservation of the WRKY DNA-binding domain in *L. ruthenicum* proteins. The selected *LrWRKY* genes exhibited discrete expression patterns during different fruit developmental stages. Interestingly, five *LrWRKYs* (-*20*, -*21*, -*28*, -*30*, and -*31*) were expressed remarkably throughout the fruit developmental stages.

**Discussion:**

Our results reveal the characteristics of the *LrWRKY* gene family, thus laying a foundation for further functional analysis of the *WRKY* family in *L. ruthenicum.*

## Introduction

WRKY transcription factors (TFs) are the 7th largest family of regulatory genes screened from higher plants (Marchive et al., 2007; Chen et al., 2018a; Chen et al., 2018b). The first cDNA encoding a WRKY protein, *SPF1*, was cloned from *Ipomoea batatas* (Ulker & Somssich, 2004; Ling et al., 2011). Since then, several members of the WRKY proteins have been identified from several plant species (Liu & Ekramoddoullah, 2009; Liu et al., 2012). To date, only two WRKY homologs in non-plant species have been described, in *Giardia lamblia* and *Dictyostelium discoideum (Wu et al., 2005)*. Notably, 72 *WRKY* members in *Arabidopsis thaliana* (Dong, Chen & Chen, 2003), 182 in *Glycine max* (Bencke-Malato et al., 2014), 59 in *Fragaria vesca* (Zhou et al., 2016), 127 in *Malus domestica* (Meng et al., 2016), 55 in *Cucumis sativus* (Ling et al., 2011), 80 in *Vitis vinifera* (Zhang & Feng, 2014), 86 in *Brachypodium distachyon* (Wen et al., 2014), 85 in *Manihot esculenta* (Wei et al., 2016), and 95 in *Daucus carota* (Li et al., 2016) have been identified. The name of the WRKY family is derived from the most common characteristic of these proteins, the WRKY domain, which is characterized by approximately 60 amino acid residues that often exhibit sequence-specific DNA-binding (Chen et al., 2018a; Chen et al., 2018b; Zhou et al., 2016). In the WRKY domain, the highly conserved heptapeptide sequence WRKYGQK is followed by a CX_4−5_ X_4_-CX_22−23_-HXH (C_2_H_2_) or CX_7_-CX_23_X_23_-HXC (C_2_HC-) type zinc-finger motif (Eulgem et al., 2000). The conserved WRKY domain may have a longer sequence, for example, WRKYGKK or WEKYGQK. It may also be replaced by WRRY, WKKY, WIKY, WSKY, WKRY, WRIC, WVKY, or WRMC. The conserved WRKY domain helps in binding of the protein to W-box elements or sugar-responsive cis-elements (SURE) in the promoter regions of target genes (Sun et al., 2003; Rushton et al., 2010). Depending on the number of WRKY domains and the zinc-finger motif, the WRKY proteins can be categorized into three main groups (1, 2, and 3) (Eulgem et al., 2000). Group 1 proteins have two WRKY domains: an N-terminal WRKY domain and a C-terminal WRKY domain, followed by a C_2_H_2_ zinc-finger motif (CX_4_ X_4_-CX_22−23_HXH). Group 2 proteins have only one WRKY domain and a C_2_H_2_ zinc-finger motif (CX_4−5_ X_4_-CX_23_-HXH); this group is further divided into five subgroups (2a to 2e) according to the phylogeny of the WRKY domain. Group 3 proteins also have a single WRKY domain and a C_2_HC zinc-finger motif (CX_7_-CX_23_ X_23_-HXC) (Eulgem et al., 2000; Rushton et al., 2010).

The *WRKY* gene family has recently gained attention for its involvement in a wide range of biological and physiological developmental processes in plants, including diverse biotic/abiotic stress responses (Li et al., 2016; Jiang et al., 2017; Yang et al., 2016). The *WRKY* gene family also plays a crucial role in the developmental and ripening processes of fruits; for example, *FvWRKY4, -46*, and *-48* in group 2c are involved in fruit development and ripening in *F. vesca* (Zhou et al., 2016). In *Ziziphus jujube ZjWRKY8, -26, -47,* and *-48* are involved in developmental process in younger fruits (Xue et al., 2019). In addition to the involvement in seed or fruit development, the *WRKY* genes also control seed pigmentation. *AtWRKY44* (transparent testa glabra, *TTG2*) controls the epidermal color of *Arabidopsis* seeds by contributing to transcriptional regulation. It directly binds the upstream regulatory region of *TT12*. *TTG1, TT2*, and *TT8* are involved in the biosynthesis of proanthocyanidins and pigmentation in *Arabidopsis* (Gonzalez et al., 2016). Moreover, *OsWRKY11* in *O. sativa* is involved in controlling flower development (Cai et al., 2014). The roles of WRKYs in plant responses to abiotic stresses such as drought, cold, salinity, heat, ultraviolet B (UV-B), osmotic stress, and ABA have been extensively documented (Luo et al., 2013; Jiang, Liang & Yu, 2012; Ren et al., 2010; Tao et al., 2011; Li et al., 2010; Liu & Guan, 2012).

*Lycium ruthenicum* Murr. is a typical native halophyte and perennial shrub distributed across arid and semi-arid regions of Asia, America, and Africa (Liu et al., 2012; Dai, Wang & Zhang, 2016). Its excellent physiological features conferring drought resistance, coupled with its salt tolerance, make it a relevant plant for land desertification control and soil salinity improvement (alkalinity) (Zheng et al., 2011). Ripe *L*. *ruthenicum* fruits are considered the highest and richest wild source of anthocyanins and are unique from a pharmaceutical point of view (Chen et al., 2013). For instance, the fruits are used to treat health conditions, including early-onset diabetes, anemia, vision problems, impotency, and lung disorders. Recently evidence indicates that these fruits can improve liver and kidney function, boost the immune system, and promote longevity (Zheng et al., 2011; Dhar et al., 2011). Consequently, worldwide economic interest in the production of *L. ruthenicum* has recently grown. *L. ruthenicum* is not only an economically and ecologically important plant but could also be a model organism for research. The *WRKY* gene family has been comprehensively studied in many plant species (Jiang, Liang & Yu, 2012; Ren et al., 2010; Tao et al., 2011; Liu & Guan, 2012), but no previous studies have described the *WRKY* genes from *L*. *ruthenicum*. Because of the importance of the *WRKY* genes in various physiological and biological plant processes, systematic study of the identification, classification, expression, and functions of *WRKY* family in the fruit development of *L. ruthenicum* is of interest.

In the present study, we identified a total of 73 potential *LrWRKY* members in *L. ruthenicum* according to a transcriptome database. Forty-four *LrWRKYs* identified with WRKY domains were selected, and the identification, classification, phylogenetic analysis, conserved motif determination, and gene expression profiling were extensively examined. The expression levels of *WRKY* genes in different fruit developmental stages were further validated using RT-qPCR. This study reveals clues to aid in the functional characterization of *LrWRKY* genes and provides a basis for further genomic studies in *L. ruthenicum*.

## Materials & Methods

### Sequence database search and identification of the LrWRKY family in *L. ruthenicum*

*A. thaliana* and *O. sativa* WRKY protein sequences were obtained from The Arabidopsis Information Resource (TAIR) database (http://www.arabidopsis.org/) and the Joint Genome Institute (JGI) database (https://phytozome.jgi.doe.gov) separately and were used for comparative analysis. To obtain a comprehensive list of LrWRKY proteins, we obtained transcriptome data from the public Sequence Read Achieve database of NCBI (https://www.ncbi.nlm.nih.gov/sra) under accession number SRR7700825 (Ma et al., 2018). To obtain the transcriptome sequence encoding WRKY proteins, we performed a Blastx search in the NCBI database. *A. thaliana* WRKY domain sequences were used as query sequences, and Blast default settings were used. The nucleotide sequences of the possible *LrWRKYs* were translated into amino acid sequences in DNAMAN 8.0 (LynnonBioSoft, Quebec, Canada) software, to examine WRKY proteins encoded by the obtained transcriptome sequences (Data S1). The Protein families database (Pfam; http://pfam.xfam.org/) was used to verify whether the putative proteins had the distinctive features of WRKY proteins. Additionally, manual examination of the conserved WRKYGQK amino acid structure at the N-terminus and the zinc-finger structure at the C-terminus of the putative WRKY domain was conducted for further confirmation (Zhang & Wang, 2005).

### Multiple sequence alignment, phylogenetic analysis, and conserved motif construction

All 44 LrWRKY proteins with WRKY domains and several selected AtWRKY proteins (AtWRKY6, -14, -25, -26, -31, -40, and -51) were used to generate multiple protein sequence alignments by using the default settings in ClustalW (Larkin et al., 2007). The 44 LrWRKY proteins with WRKY proteins from two model plant species, *A*. *thaliana* (72) and *O. sativa* (73) (Data S2), were used for phylogenetic tree construction in MEGA 5.0 software (Tamura et al., 2011) with the maximum likelihood method and 1000 bootstrap replications. The conserved motifs in LrWRKY proteins were determined with Multiple Expectation Maximization for Motif Elicitation (MEME) suite version 5.1.1 (http://meme-suite.org/doc/cite.html), and five motifs were searched.

### Plant materials

*L. ruthenicum* fruit samples were collected from Shizuishan in Ningxia, China (38°56.799′ N, 106°24.711′ E, 1088 H) in three developmental stages, Rs1 (green ripeness stage), Rs2 (veraison ripeness stage), and Rs3 (complete ripeness stage), on the basis of the descriptions of Ma et al. (2018) and Guillaumie et al. (2011). Biological replicates of the material comprised three shrubs in each developmental stage. The fruit samples from the three shrubs were collected, immediately frozen in liquid nitrogen, and stored at −80 °C until total RNA isolation.

### RNA extraction, cDNA synthesis, and RT-qPCR analysis

We extracted total RNA from nine samples (three biological replicates per treatment) by using a TransZol Up Plus RNA Kit (Lot# M31018) conferring to the manufacturer’s instructions. The RNA quantity and quality were determined with a TGen Spectrophotometer (TianGen), on the basis of the A260 nm/A280 nm and A260 nm/A230 nm ratios. An *Evo* M-MLV RT Kit AG11705 (Accurate Biotechnology) was used to reverse transcribe the total RNA into cDNA and remove any genomic DNA from the cDNA before RT-qPCR analysis, following the manufacturer’s instructions.

For the RT-qPCR analysis, primers were designed on the basis of the RNA sequences obtained from the NCBI SRA (Ma et al., 2018) in Primer 5 software and synthesized by TsingKe Biological Technology Co., Ltd. (Xi’an, China) (Data S3). To better understand the *LrWRKY* gene expression during different fruit developmental stages, we selected 23 genes with FPKM values >5 from the 44 genes with WRKY domains for RT-qPCR analysis. The *LrEF1-a* gene (JX427553) was used as a reference gene. We examined three biological replicates and performed triplicate quantitative analyses; each replicate was analyzed on the basis of 0.5 µl of each cDNA dilution with a Heiff^®^ q-PCR SYBR^®^ Green Master Mix kit (Yeasen Biotech Co., Ltd) according to the manufacturer’s protocol. The RT-qPCR analysis was performed with a QuantStudio™ 5 Real-Time PCR Instrument (ABI) (Data S4). The *LrWRKY* gene relative expression was calculated according to Livak & Schmittgen (2001).

A heatmap of all 44 *LrWRKY* gene expression patterns was constructed during fruit development and ripening stages, on the basis of FPKM values (Data S5) in TBtools software (https://github.com/CJ-Chen/TBtools) (Chen et al., 2018a; Chen et al., 2018b).

### Data analysis

All reported gene expression levels are presented as means ± SE (*n* = 3). The significance level among means was analyzed with Duncan’s multiple range tests (*p* ≤ 0.05) after one-way ANOVA analysis in SPSS statistical software (Ver. 22.0, SPSS Inc., Chicago, IL, USA).

## Results

### Identification of *LrWRKY* genes

We searched 43,573 unigenes for all possible *LrWRKY* genes from the transcriptome data of *L. ruthenicum* and obtained a total of 73 best hits, which were validated by BLAST via the NCBI database. We use the prefix *“Lr”* for *L. ruthenicum* and assigned the WRKY numbering according to the order of the annotated sequences from the database, as CL10207Contig1 (*LrWRKY1*) to CL8621Contig1 (*LrWRKY73*), respectively (Data S6). The lengths of members of the *WRKY* family in *L*. *ruthenicum* ranged from 5207 aa (*LrWRKY3*) to 485 aa (*LrWRKY67*). The open reading frames (ORF) of all members could not be predicted, due to the limitations of the sequencing technique.

### Phylogenetic analysis of *LrWRKY* genes

**Figure 1:**
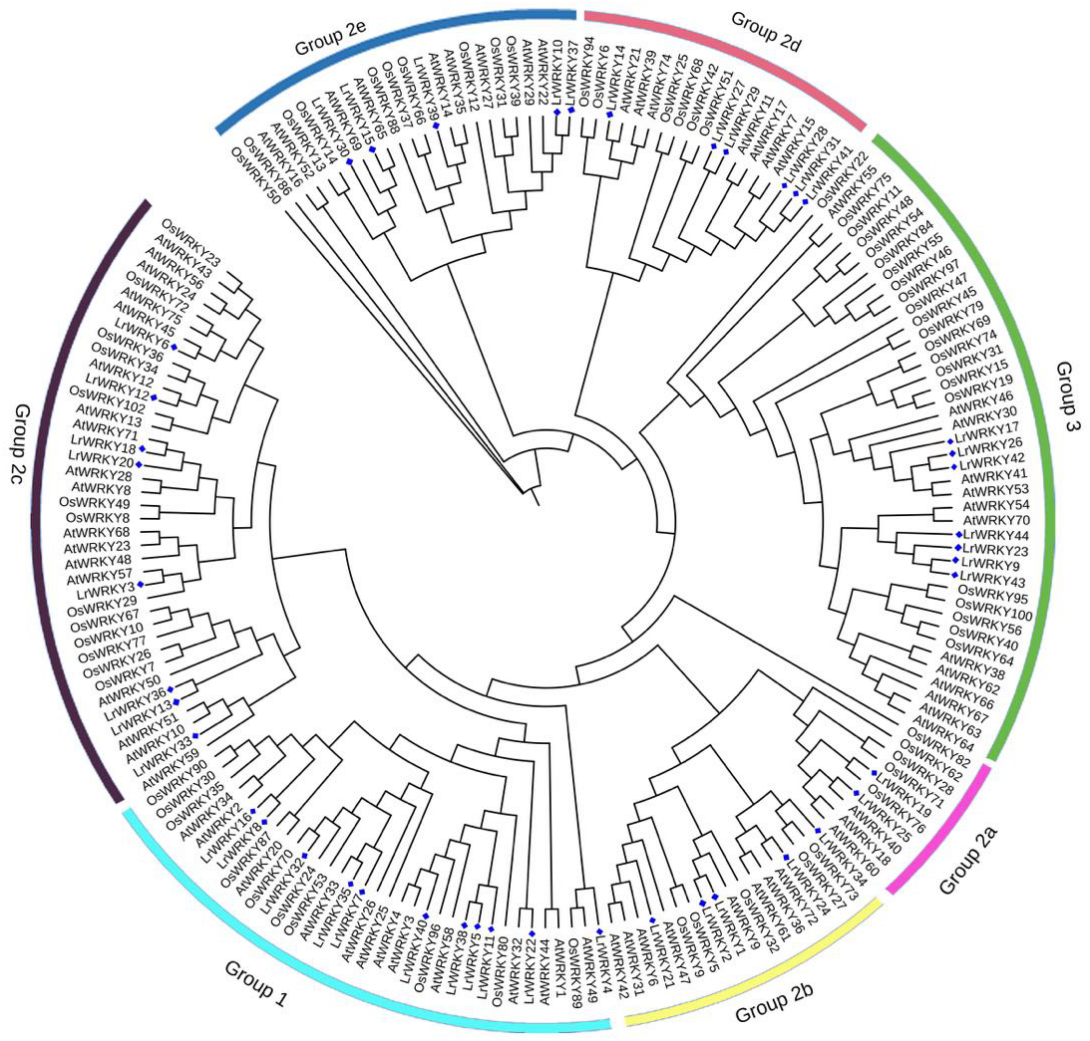
Phylogenetic tree according to WRKY domain sequences from *L. ruthenicum, O. sativa and A. thaliana*. Consistency of the predicted tree was tested using the Maximum Likelihood method and bootstrapping with 1,000 replicates in MEGA 5. The different colored arcs indicate different groups or subgroups and the blue diamonds represent LrWRKYs.

Phylogenetic analysis of plant *WRKY* genes is an effective way to understand the functions of uncharacterized WRKY members according to their evolutionary history and sequence similarity. Therefore, the 44 LrWRKYs identified to have WRKY domains were used to construct a phylogenetic tree. The phylogenetic analysis revealed that the LrWRKY proteins could be categorized into three main groups corresponding to groups 1, 2, and 3 in *A. thaliana* (Eulgem et al., 2000). Among the 44 LrWRKY proteins, 10 were classified into group 1 (representing 23%) consisting of N-terminal and C-terminal subgroups, 26 LrWRKY proteins were assigned to group 2 (representing 59%), and the remaining 8 LrWRKY proteins were classified in group 3 (representing 18%) (Fig. 1). Group 1 *WRKY* family members usually have two WRKY domains and a C_2_H_2_-type zinc-finger motif (CX_4_X_4_-CX_22−23_-HXH), but few *LrWRKYs* from group 1 contained only one WRKY domain (LrWRKY8, -11, -32, -35, and -38). LrWRKY proteins in group 2 had a single WRKY domain and a C_2_H_2_-type zinc-finger motif (CX_4−5_ X_4_-CX_23_-HXH), which was further subdivided into five distinct subgroups (2a to 2e): two LrWRKYs belonged to 2a, four LrWRKYs belonged to group 2b, eight belonged to group 2c, six belonged to group 2d, and five belonged to group 2e, according to their phylogeny. LrWRKYs from group 3 were characterized by a C_2_HC-type zinc-finger motif (CX_7_-CX_23_ X_23_-HXC) (Table 1).

**Table 1:** LrWRKYs grouping, zinc-finger structure and domain pattern.

**Gene name**	**Group classification**	**Zinc finger**	**Domain pattern**	**Gene name**	**Group classification**	**Zinc finger**	**Domain pattern**
LrWRKY1	2b	C_2_H_2_	N	LrWRKY23	3	C_2_HC	CX_7_- CX_23_X_23_- HXC
LrWRKY2	2b	C_2_H_2_	N	LrWRKY24	2b	C_2_H_2_	CX_5_- CX_23_- HXH
LrWRKY3	2c	C_2_H_2_	CX_4_- CX_23_- HXH	LrWRKY25	2a	C_2_H_2_	CX_5_- CX_23_- HXH
LrWRKY4	3	C_2_H_2_	CX_7_- CX_23_X_23_- HXC	LrWRKY26	3	C_2_HC	CX_7_- CX_23_X_23_- HXC
LrWRKY5	1	C_2_H_2_	CX_4_X_4_- CX_22−23_- HXH	LrWRKY27	2d	C_2_H_2_	CX_5_- CX_23_- HXH
LrWRKY6	2c	C_2_H_2_	CX_4_- CX_23_-HXH	LrWRKY28	2d	C_2_H_2_	CX_5_- CX_23_- HXH
LrWRKY7	1	C_2_H_2_	CX_4_X_4_- CX_22−23_- HXH	LrWRKY29	2d	C_2_H_2_	CX_5_- CX_23_- HXH
LrWRKY8	1	C_2_H_2_	CX_4_X_4_- CX_22−23_- HXH	LrWRKY30	2e	C_2_H_2_	CX_5_- CX_23_- HXH
LrWRKY9	3	C_2_HC	CX_7_- CX_23_X_23_- HXC	LrWRKY31	2d	C_2_H_2_	CX_5_- CX_23_- HXH
LrWRKY10	2e	C_2_H_2_	CX_5_X_4_- CX_23_- HXH	LrWRKY32	1	C_2_H_2_	CX_4_- CX_22−23_- HXH
LrWRKY11	1	C_2_H_2_	CX_4_X_4_- CX_22−23_- HXH	LrWRKY33	2c	C_2_H_2_	CX_4_- CX_23_- HXH
LrWRKY12	2c	C_2_H_2_	CX_4_- CX_23_- HXH	LrWRKY34	2b	C_2_H_2_	CX_5_- CX_23_- HXH
LrWRKY13	2c	C_2_H_2_	CX_4_- CX_23_- HXH	LrWRKY35	1	C_2_H_2_	CX_4_- CX_22−23_HXH
LrWRKY14	2d	C_2_H_2_	CX_5_X_4_- CX_23_- HXH	LrWRKY36	2c	C_2_H_2_	CX_4_- CX_23_-HXH
LrWRKY15	2e	C_2_H_2_	CX_5_X_4_- CX_23_- HXH	LrWRKY37	2e	C_2_H_2_	CX_5_- CX_23_- HXH
LrWRKY16	1	C_2_H_2_	CX_4_X_4_- CX_22−23_- HXH X_22−23_	LrWRKY38	1	C_2_H_2_	N
LrWRKY17	3	C_2_HC	CX_7_- CX_23_X_23_- HXC	LrWRKY39	2e	C_2_H_2_	CX_5_- CX_23_-HXH
LrWRKY18	2c	C_2_H_2_	CX_4_- CX_23_- HXH	LrWRKY40	1	C_2_H_2_	CX_4_- CX_22−23_- HXH
LrWRKY19	2a	C_2_H_2_	CX_5_X_4_- CX_23_- HXH	LrWRKY41	2d	C_2_H_2_	CX_5_- CX_23_- HXH
LrWRKY20	2c	C_2_H_2_	CX_4_- CX_23_- HXH	LrWRKY42	3	C_2_HC	CX_7_- CX_23_X_23_- HXC
LrWRKY21	2b	C_2_H_2_	CX_5_X_4_- CX_23_- HXH	LrWRKY43	3	C_2_HC	CX_7_- CX_23_X_23_- HXC
LrWRKY22	1	C_2_H_2_	CX_4_X_4_- CX_22−23_- HXH	LrWRKY44	3	C_2_HC	CX_7_- CX_23_X_23_- HXC

**Notes.**

N, missen domain pattern.

### Multiple sequence alignment of *LrWRKY* genes

**Figure 2:**
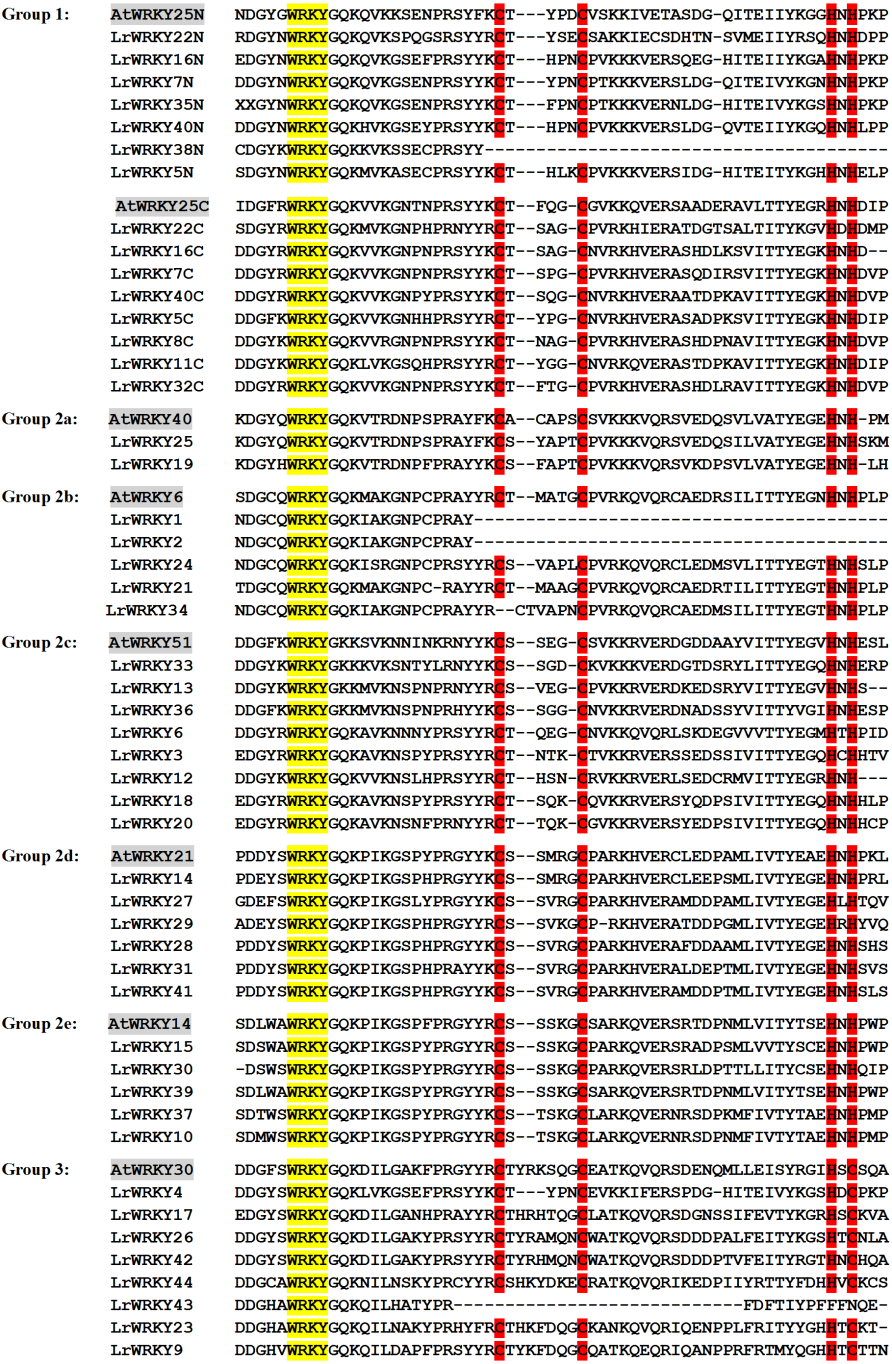
Multiple sequence alignment of the 44 LrWRKY proteins and selected *A. thaliana* proteins. ‘N’ and ‘C’ indicate the N-terminal and C-terminal WRKY domains. The conserved WRKY amino acid signature and the amino acids forming the zinc-finger motifs are highlighted in yellow and red, missing amino acids are represented by dashes.

The evolutionary relationship of the *LrWRKY* genes was analyzed through multiple sequence alignment of their conserved WRKY domains. For examination of the core domains, WRKY domains of AtWRKY proteins were selected (AtWRKY6, −14, −21, −25, −30, −40, and −51) from each group or subgroup as representatives for further comparison. The 44 LrWRKY proteins contained the highly conserved WRKY domains, and 41 were discovered to have the conserved heptapeptide sequence of WRKYGQK, whereas three members of group 2c (LrWRKY13, −33 and −36) differed by one amino acid, with the glutamine (Q) substituted by a lysine (K), thus forming WRKYGKK (Fig. 2). For group 1 members, the two WRKY domains, which were denoted group 1 N-terminal and group 1 C-terminal, clustered into two separate groups because of divergence in their sequences. We discovered a CX_4_-CX_22_-HXH zinc-finger structure in the N-terminal subgroup and a CX_4_-CX_23_-HXH structure in the C-terminal subgroup.

**Figure 3:**
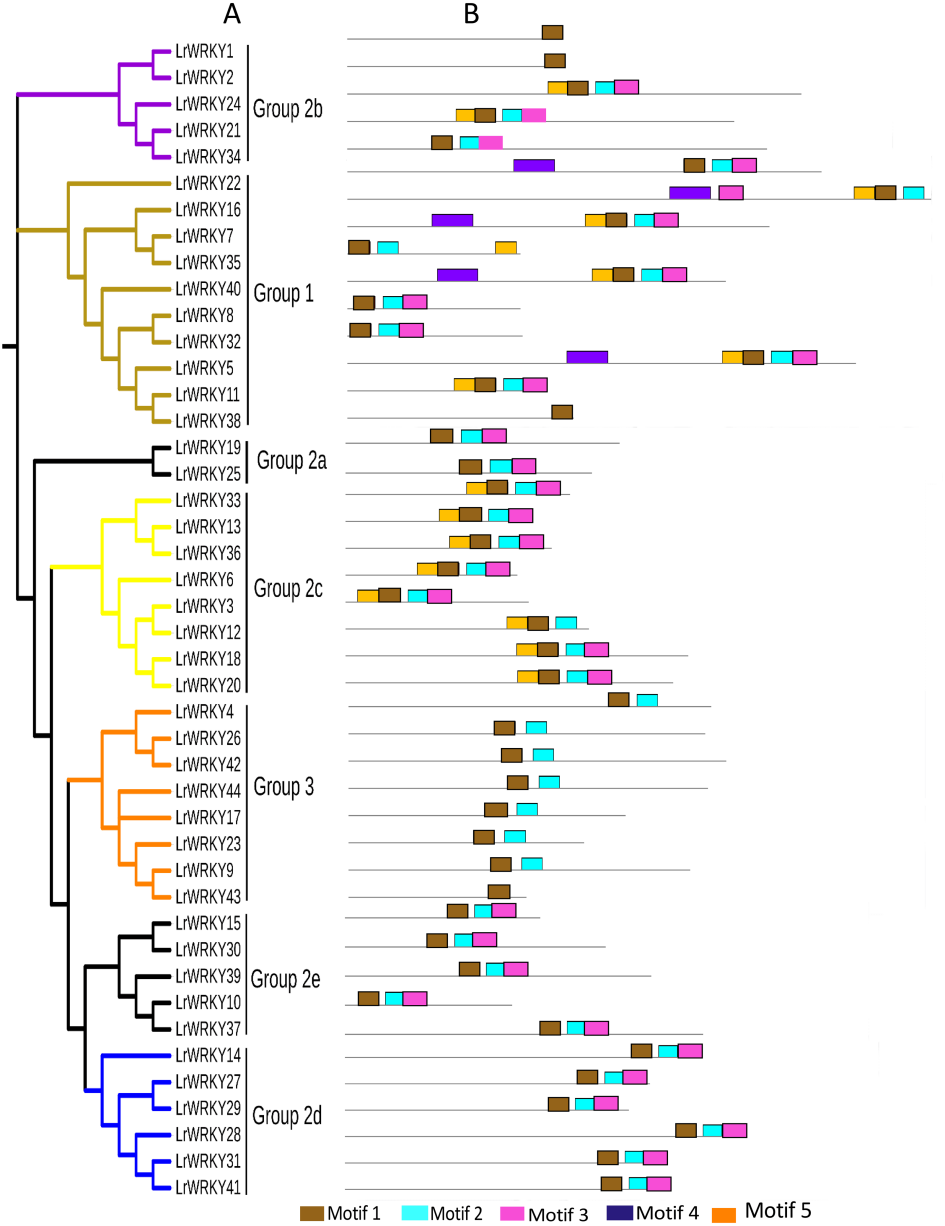
Phylogenetic relations and conserved protein motifs in *L. ruthenicum*. (A) The unrooted phylogenetic tree was constructed according to WRKY proteins sequences of *L. ruthenicum* with 1,000 bootstrap replicates. Details of groups are shown in different colors. (B) The motif composition of LrWRKY protein were identified using MEME. The different colored boxes represent different motifs and their position in each WRKY sequence. Each motif is indicated by a colored box indicated in the key at the bottom.

### Conserved motif analysis of LrWRKYs proteins

The conserved motifs of *L. ruthenicum* were examined with MEME, as described in the methods, to better understand the relationship and diversity of LrWRKY protein motif compositions. The results revealed that motifs 1 and 4 were WRKY DNA-binding motifs that encompassed the conserved heptapeptide WRKYGQK domain. The motif 4 WRKY domain belonged to the N-terminal domain, whereas motif 1 belonged to the C-terminal domain (Fig. 3, Table 2). Interestingly, motif 5 had the largest number of amino acid sequences and was present in groups 1, 2b, and 2c, whereas motif 3 had the smallest number of protein sequences but was present in all groups/subgroups except group 3. Most of the LrWRKY proteins contained at least two motifs. LrWRKY members belonging to the same group were discovered to share analogous motif compositions; for instance, motif 4 was exceptional to group 1 members. Groups 2a, 2e, and 2d contained three motifs (-1, -2, and -3) whereas group 2b and 2c contained four motifs (-1, -2, -3, and -5), except for LrWRKY1 and -2 which contained only motif 1. In addition, LrWRKY12 and -34 belonged to group 2b and 2c but did not contain motif 5. Group 3 contained motifs 1 and -2, except for LrWRKY43, which contained only motif 1. Comparisons between randomly selected AtWRKY members with their LrWRKY orthologs revealed that AtWRKY6, -14, -21, -25, -30, -40, and -51 shared the same motif compositions with their prospective LrWRKY group members (Data S7). Only LrWRKY1, -2, -38, and -43 motif compositions differed from the selected AtWRKY members.

**Table 2:** Analysis and conserved motifs determination in LrWRKY proteins.

**Motif**	**E value**	**Width**	**Motif sequence**
**1**	8.9e−762	21	GYSWRKYGQKPVKGSPYPRSY
**2**	2.9e−500	21	CPVRKQVERSSEDPSMLITTY
**3**	5.8e−114	11	EGEHNHPVPAA
**4**	2.0e−112	21	KKVRKPRVAVRTRSEVDILDD
**5**	8.4e−104	41	DGYNWRKYGQKQVKGSEYPRSYYKCTHPNCP VKKKVERSLD

### Expression analysis of *LrWRKYs* in different developmental stages of fruits

**Figure 4:**
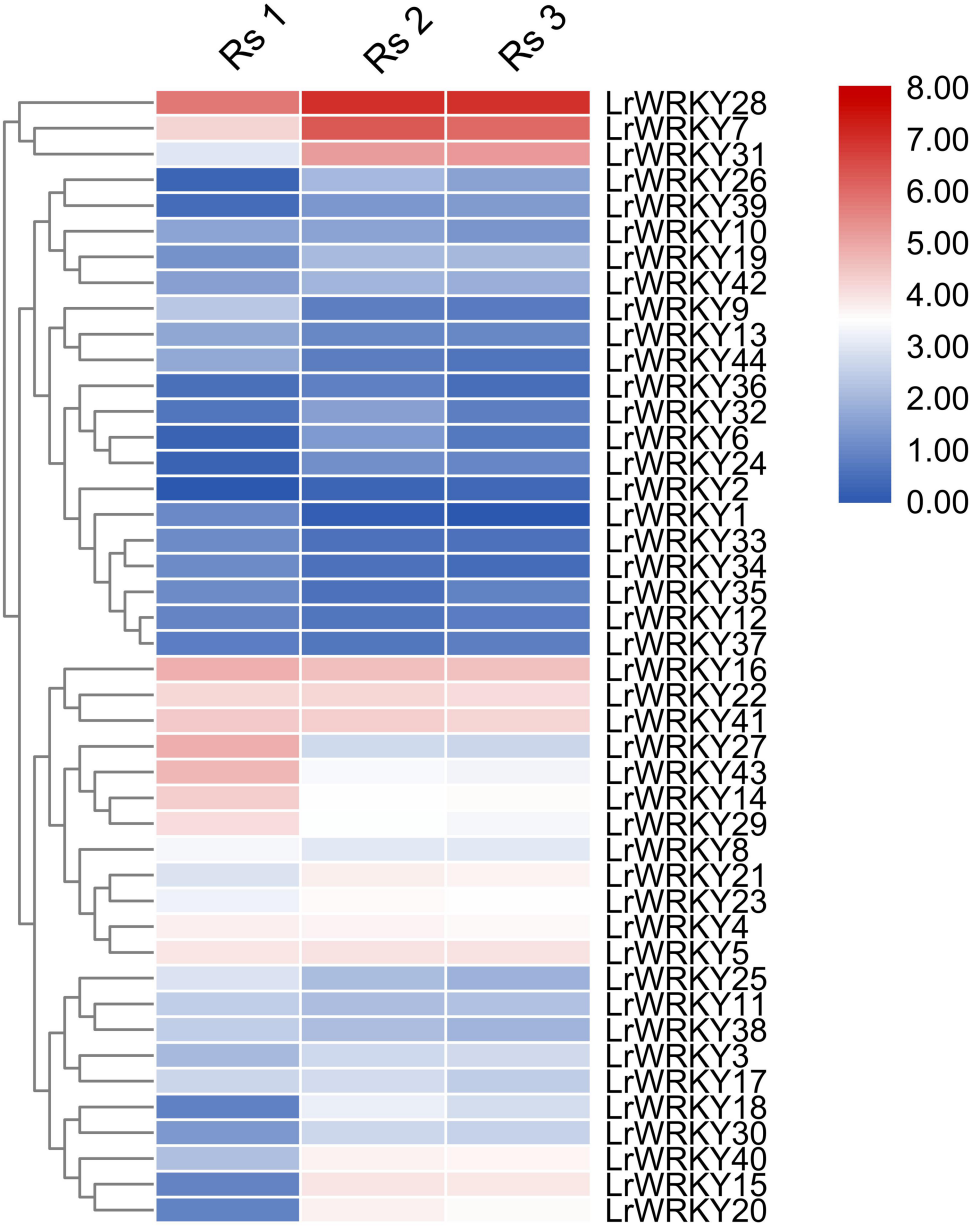
Heatmap representation of the *LrWRKY* genes during fruit developmental stages of *L. ruthenicum*. The expression profiles of the 44 *LrWRKY* genes were based on FPKM-values. Red and blue boxes indicate high and low expression levels for each gene.

Expression profiles of the 44 *LrWRKY* genes were evaluated in three different fruit developmental stages (Rs 1, Rs 2, and Rs 3). The heatmap illustration of expression profiles of *LrWRKY* genes is shown in Fig. 4. The expression profiles revealed that all 44 *LrWRKYs* exhibited discrete expression during fruit development. Twenty-one *LrWRKYs* showed insignificant expression in all developmental stages of the fruit, and the FPKM values were <5 in all three stages. The remaining 23 members of *LrWRKYs* showed relatively higher expression, with FPKM >5 in at least one of the fruit developmental stages. Six *LrWRKY* genes exhibited markedly high expression patterns across all fruit developmental stages, with FPKM values >20. Interestingly, we also discovered that some *WRKY* genes classified in the same groups/subgroups had differential expression. For example, group 1, group 2d, *LrWRKY3*, and *-20* from group 2c exhibited substantial expression with high FPKM values. However, group 2b members were weakly expressed in almost all developmental stages. The lowest FPKM value recorded in group 2d was 5.3, whereas some genes had FPKM values as high as 130.1 (Fig. 4, Data S4).

**Figure 5:**
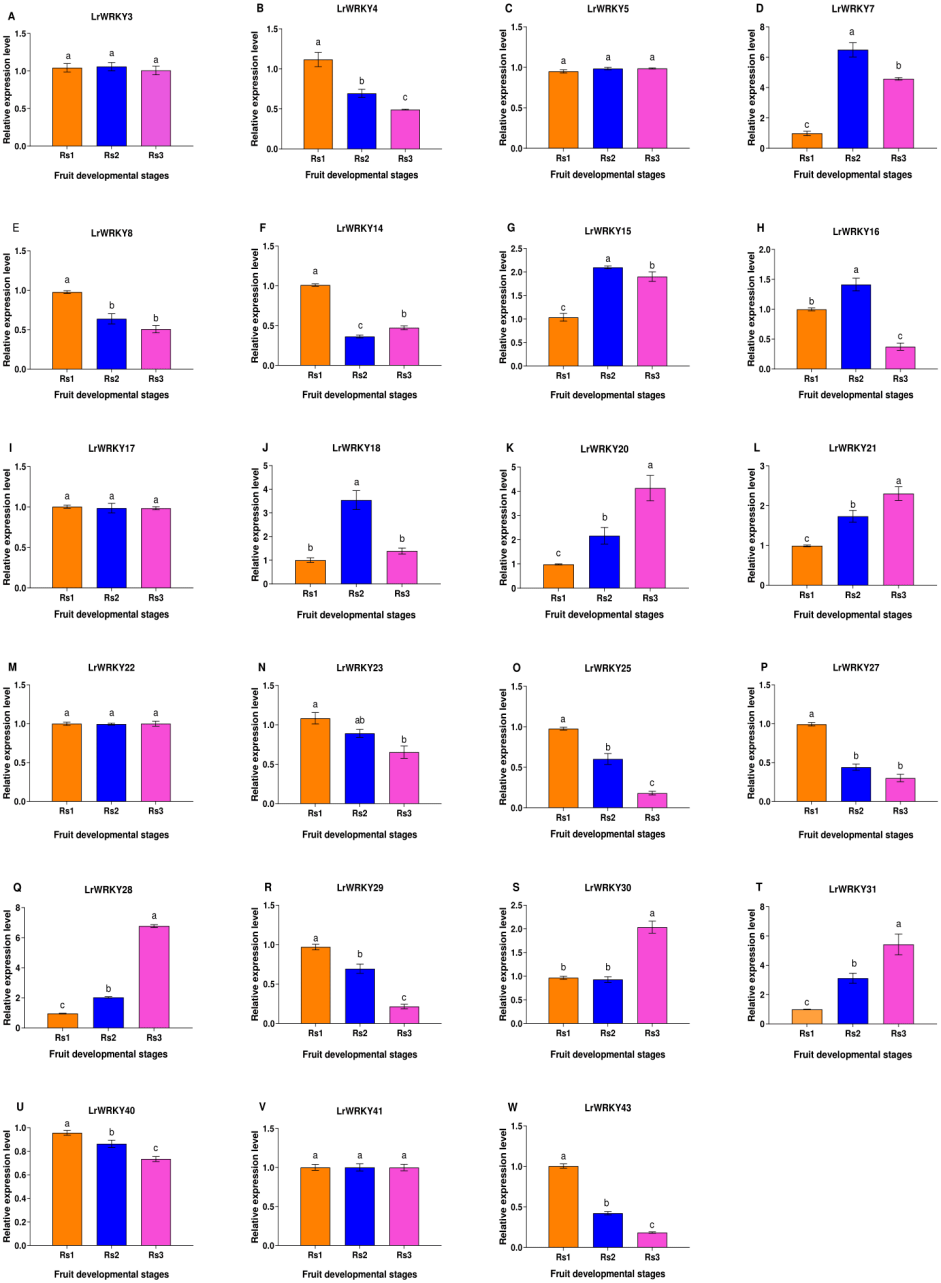
RT-qPCR validation of twenty-three selected *LrWRKYs* expression levels during fruit developmental stages. The Rs1 represent green ripeness stage, Rs2 represent veraison ripeness stage, and Rs3 represent complete ripeness stage. The name of the gene is written on top of each bar diagram; (A) LrWRKY3, (B) LrWRKY4, (C) LrWRKY5, (D) LrWRKY7, (E) LrWRKY8, (F) LrWRKY14, (G) LrWRKY15, (H) LrWRKY16, (I) LrWRKY17, (J) LrWRKY18, (K) LrWRKY20, (L) LrWRKY21, (M) LrWRKY22, (N) LrWRKY23, (O) LrWRKY25, (P) LrWRKY27, (Q) LrWRKY28, (R) LrWRKY29, (S) LrWRKY30, (T) LrWRKY31, (U) LrWRKY40, (V) LrWRKY41, (W) LrWRKY43. The different letters indicate significant differences among developmental stages (*p* < 0.05).

To gain a deeper knowledge of the gene expression patterns and to validate the *LrWRKY* genes, we performed RT-qPCR analysis of 23 selected genes. *LrWRKYs* exhibited different expression patterns in Rs 1, Rs 2, and Rs 3 (Fig. 5). Some *LrWRKYs* members maintained stable expression under different ripening stages, for example, *LrWRKY3*, -*5*, -*17*, -*22*, and -*41*. A total of nine *LrWRKYs* (-*4*, -*8*, -*14*, -*23*, -*25*, -*27*, -*29*, -*40*, and -*43*) decreased significantly from Rs 1 to Rs 3, among which *LrWRKY 8*, -*23*, and -*27* maintained stable expression in Rs 2 and Rs 3. However, five *LrWRKYs* (-*20*, -*21*, -*28*, -*30*, and -*31*) continuously increased from Rs 1 to Rs 3, except for *LrWRKY 30*, which maintained stable expression in RS 1 and RS 2. In addition, three *LrWRKYs* increased first and peaked in RS 2, as compared with RS 1, and then decreased in RS 3.

## Discussion

The *WRKY* gene family is one of the major families of TFs with crucial roles in various plant physiological processes (Huang et al., 2012; Huang et al., 2015; He et al., 2012). Genome-wide analysis, including identification and expression profiling of *WRKY* genes in *V. vinifera* (Wang et al., 2014), *F. vesca* (Zhou et al., 2016), and *A. thaliana* (Miao et al., 2004), has been broadly investigated. Because of technical limitations, the reference genome database of *L. ruthenicum* is not available. In this present study, the *WRKY* family analysis in *L. ruthenicum* was mainly based on transcriptome data, and a total of 73 possible *WRKY* gene members were identified. Forty-four *LrWRKY* genes with the conserved WRKY domain were identified. Our results provided the first systematically identified set of *WRKY* family members in *L. ruthenicum*.

After construction of the evolutionary tree and multiple sequence alignment based on *L. ruthenicum* WRKY conserved domains, we discovered that the 44 *LrWRKYs* could be categorized into three major groups, as described earlier in *A. thaliana* by Eulgem et al. (2000). The N-terminal and C-terminal WRKY domains in group 1 were assembled into different groups, thus potentially revealing separate evolution of the two domains. Previous studies have described group 2 *WRKY* genes as the group with the most members, for example, *A. thaliana* (Eulgem et al., 2000), *B. distachyon* (Wen et al., 2014), *Caragana intermedia* (Wan et al., 2018), and *M. esculenta* (Wei et al., 2016). Our study also revealed that group 2 *LrWRKY* genes had the highest proportion of members (59%), thus suggesting the activity of group 2 during the evolutionary processes and indicating that this group might have regulatory functions in fruit development and ripening. Group 3 showed the smallest number of *LrWRKY* genes (18%) among the three groups, possibly because of transcriptome data limitations or underestimation during our analysis. The WRKY TFs have multiple diverse functions in plant growth and various stress responses, and *WRKY* genes within the same group or subgroup usually retain similar regulatory functions (Chen et al., 2018a; Chen et al., 2018b). Group 1 *WRKY* genes have been reported to participate in a wide range of plant growth and development processes. For instance, *AtWRKY2, -26, -34,* and *-44* in group 1 are involved in seed germination, post-germination growth, leaf senescence, and root hair growth in *Arabidopsis* (Guan et al., 2014; Li et al., 2017; Gonzalez et al., 2016), and they share a similar homologous relationship with their *L. ruthenicum* counterparts (*LrWRKY4, -5, -7, -8, -11 -16, -22, -32, -35, -38,* and *-40*), thus suggesting related developmental roles among the genes. Most of the group 2a subfamily genes are involved in stress signaling, and group 2a genes in *L. ruthenicum* may share similar functions. Some members of group 2c (*AtWRKY10, -28, -57, -75,* and *OsWRKY72*) in *A. thaliana* and *O. sativa* have been reported to be involved in salt stress, flowering, seed development, and coloration (Chen et al., 2018a; Chen et al., 2018b); these members have homologous relationships with *LrWRKY (-3, -6, -12, -13, -18, -20, -33,* and *-36)* in group 2c. In addition, *ZjWRKY26; VvWRKY14, -19, -52;* and *FvWRKY4, -46,* and *-48,* belonging to group 2c, have been reported to be mainly involved in the developmental process of fruits in *Z. jujube* (Xue et al., 2019), *V. vinifera* (Wang et al., 2014), and *F. vesca* (Zhou et al., 2016) respectively. Similarly, the group 2d subfamily genes are involved in the regulation of growth and development. Among the WRKY groups, group 3 genes are considered to have evolved the most, and they appear to play a crucial role in plant adaptation and development (Tao et al., 2018). In *A. thaliana*, group 3 members (*AtWRKY46, -54,* and *-70*) are involved in brassinosteroid signaling and regulate both osmotic stress and plant development (Chen et al., 2017). Thus, the specific functions of WRKYs might provide clues regarding the same homologs in *LrWRKYs* for further functional identification.

Nevertheless, the conserved heptapeptide WRKY domain WRKYGQK is the most common characteristic of WRKY proteins, although this sequence is sometimes substituted by WRKYGKK through mutation (Rushton et al., 2010). Our analysis uncovered substitution of some WRKYGQK sequences (*LrWRKY13, -33,* and *-36*) in group 2c with a WRKYGKK sequence, a result similar to findings describing the substitution of glutamine (Q) by lysine (K) in *A. thaliana* (*AtWRKY51, -52,* and *-59*) (Eulgem et al., 2000), *C. intermedia* (*CiWRKY2, -41, -50,* and -*51*) (Wan et al., 2018), and *Panicum miliaceum* (*PmWRKY2, -15, -23, -24,* and *-28*) (Yue et al., 2016). According to Van Verk et al. (2008), the deviation of the WRKYGQK motif might affect DNA binding activity; therefore, *LrWRKY13 -33*, and -*36* genes are recommended for further investigation in studies of their functions and binding specificities. This study also established that the C_2_HC zinc-finger was present in only group 3 WRKY domains, whereas C_2_H_2_ was found in group 1 and group 2 domains; this result confirmed similar reports in *A. comosus* (Tao et al., 2011), *C. sativus* (Ling et al., 2011), and *A. thaliana* (Eulgem et al., 2000). However, this finding differs from those from an earlier report in *O. sativa* (Wu et al., 2005), indicating that the zinc-finger structure C_2_HC and C_2_H_2_ concomitantly present in the N-terminus in group 1N. Group 1 proteins usually contain two conserved WRKY domains, but *LrWRKY8, -11, -32, -35,* and *-38* group 1 proteins with a single conserved WRKY domain. This finding may be a result of the limitations of the transcriptome data.

The analysis of motif composition revealed that LrWRKYs share a similar motif composition within the same group or subgroup. Of note, AtWRKY6 -14 -21 -25 -30 -40, and -51 share similar motifs (Data S7) with their *L. ruthenicum* WRKY orthologs, thus presenting clues regarding their regulatory functions. Although the functions of the major motifs in LrWRKYs remain undefined, LrWRKYs with the same conserved motifs may have similar functions. The presence of all five motifs identified in LrWRKYs confirms their conserved nature in this study.

Many studies have shown that WRKY function in regulating plant growth and development, while also playing essential roles in plant adaptability to stress (Chen et al., 2018a; Chen et al., 2018b). *L. ruthenicum* is widely useful as an economically important shrub with fruits rich in anthocyanins. Because of the potential roles of *WRKY* genes in the developmental stages of fruit in *L. ruthenicum*, we analyzed the expression patterns of *LrWRKY* genes, on the basis of the available transcriptome data, and performed RT-qPCR validation. We identified 44 *LrWRKYs* showing different expression patterns in the heatmap and inferred their different roles in controlling fruit development. Some *LrWRKY* genes, for instance, *LrWRKYs 3 -5 -17 -22*, and *-41*, maintained stable expression under different developmental stages. Additionally, the expression of *LrWRKYs -4 -8 -14 -23 -25 -27 -29 -40*, and *-43* decreased significantly during fruit development, similarly to observations in *RcWRKY21* and *-32* in *Ricinus communis* (Li et al., 2012). Moreover, *LrWRKYs -20 -21 -28 -30*, and *-31* showed continuous significant increases in expression from green ripening to complete ripening during fruit development. Comparatively, *AtWRKY10* and *-44*, and *OsWRKY7* (in *A. thaliana* and *O. sativa*, respectively) are also highly expressed during seed/fruit development, biosynthesis of proanthocyanidins, and seed pigmentation (Zhang & Feng, 2014; Gonzalez et al., 2016; Luo et al., 2005), thus implicating similar functions in *L. ruthenicum*. In addition, *Z. jujube WRKY8 -26 -47*, and *-48* and *F. vesca WRKY4 -46*, and *-48* are expressed significantly in fruits, and their expression increases continuously from younger fruits to mature fruits (Zhou et al., 2016; Wang et al., 2014). Finally, four *LrWRKYs* (*-7 -15 -16*, and *-18*) increased first in Rs2 and then decreased in Rs3; likewise, in seed development in *Jatropha curcas*, three genes (*JcWRKY14 -22*, and *-30*) are also expressed at higher levels in the maturation stage (S2) than in the early development stage (Xiong et al., 2013), thus suggesting a putative role in regulating fruit development in *L. ruthenicum*. Nevertheless, more in-depth study is necessary to comprehensively verify the functions of all the *LrWRKY* genes.

## Conclusions

In the present study, a total of 73 WRKY members were identified in *L. ruthenicum* according to the transcriptome data. Forty-four proteins were identified with the WRKY domain and, on the basis of their amino acid sequences, were divided into three major groups with several subgroups, in accordance with those in other plant species. All 44 LrWRKY proteins contained one or two conserved WRKY domains and a zinc-finger structure. The conserved motif prediction revealed that the WRKY DNA-binding domain has been conserved in *L. ruthenicum* proteins. Phylogenetic and gene expression analysis provided in-depth knowledge of the important functions of WRKYs during fruit development and ripening. These results may serve as a foundation enabling further genomic and functional analysis of the WRKY family in *L. ruthenicum*.

##  Supplemental Information

10.7717/peerj.10207/supp-1 Supplemental Information 1 Amino acid sequence of the predicted 73 LrWRKY proteins

10.7717/peerj.10207/supp-2 Supplemental Information 2 The WRKY protein database IDs for *A. thaliana* and *O. sativa.*

10.7717/peerj.10207/supp-3 Supplemental Information 3 The primer sequences used for the RT-qPCR analysis of the 23 *LrWRKYs*

10.7717/peerj.10207/supp-4 Supplemental Information 4 The raw data for 23 selected *LrWRKYs* RT-qPCR analysis

10.7717/peerj.10207/supp-5 Supplemental Information 5 FPKM values for all the 44 *LrWRKYs* with conserved WRKY domain

10.7717/peerj.10207/supp-6 Supplemental Information 6 Gene annotation IDs and predicted gene length information of the 73 *WRKY* genes identified in *L. ruthenicum.*

10.7717/peerj.10207/supp-7 Supplemental Information 7 Overview of WRKY protein conserved motifs from LrWRKY and AtWRKY proteins

## Additional Information and Declarations

### Data Availability

The following information was supplied regarding data availability: Raw data is available in the Supplemental Files.
